# Adaptive Postural Control for Joint Immobilization during Multitask Performance

**DOI:** 10.1371/journal.pone.0108667

**Published:** 2014-10-17

**Authors:** Wei-Li Hsu

**Affiliations:** 1 School and Graduate Institute of Physical Therapy, College of Medicine, National Taiwan University, Taipei, Taiwan; 2 Physical Therapy Center, National Taiwan University Hospital, Taipei, Taiwan; Emory University School Of Medicine, United States of America

## Abstract

Motor abundance is an essential feature of adaptive control. The range of joint combinations enabled by motor abundance provides the body with the necessary freedom to adopt different positions, configurations, and movements that allow for exploratory postural behavior. This study investigated the adaptation of postural control to joint immobilization during multi-task performance. Twelve healthy volunteers (6 males and 6 females; 21–29 yr) without any known neurological deficits, musculoskeletal conditions, or balance disorders participated in this study. The participants executed a targeting task, alone or combined with a ball-balancing task, while standing with free or restricted joint motions. The effects of joint configuration variability on center of mass (COM) stability were examined using uncontrolled manifold (UCM) analysis. The UCM method separates joint variability into two components: the first is consistent with the use of motor abundance, which does not affect COM position (V_UCM_); the second leads to COM position variability (V_ORT_). The analysis showed that joints were coordinated such that their variability had a minimal effect on COM position. However, the component of joint variability that reflects the use of motor abundance to stabilize COM (V_UCM_) was significant decreased when the participants performed the combined task with immobilized joints. The component of joint variability that leads to COM variability (V_ORT_) tended to increase with a reduction in joint degrees of freedom. The results suggested that joint immobilization increases the difficulty of stabilizing COM when multiple tasks are performed simultaneously. These findings are important for developing rehabilitation approaches for patients with limited joint movements.

## Introduction

The human body consists of a large number of bones and muscles. Therefore, the neuromuscular system displays substantial redundancy at the joint and muscle level. Since the neuromuscular system contains numerous degrees of freedom (DOFs) [1], coordination among these DOFs is necessary to the accomplishment of a desired motor goal [1], [2]. It is unclear how many DOFs are coordinated to achieve various motor tasks efficiently and effectively. Thus, many human movement coordination studies have focused on the principles underlying the organization of neuromuscular DOFs [3]–[5].

The fact that the neuromuscular system is redundant leads to the possibility of flexible coordination strategies to manage unexpected perturbations and the need to control multiple tasks simultaneously [6], [7]. The flexibility provided by the large number of neuromuscular DOFs is an advantage, leading to the recent characterization of motor redundancy as motor abundance [8]. Flexible patterns of even small joint motions may help maintain an effective body schema for sensory estimation [9], [10] and responding to unexpected postural disturbances [11], [12]. Moreover, studies have shown that the ability to utilize the motor abundance decreased in aging adults [13]. Whether the use of motor abundance during multi-task performance is compromised when joints are constrained needs to be investigated.

Joint restriction constrains the ability to flexibly coordinate the joints, and might lead to reduced balance stability. This effect may increase when people are required to perform multiple motor tasks while standing. The whole body’s center of mass (COM) is often considered the main objective of central nervous system (CNS) control [14], [15]. Upright human posture is inherently unstable because of the difficulty in maintaining a high COM position within a relatively small base of support at the feet. The COM might shift when performing a movement with the arms while standing, and consequently could result in activity adjustment of postural muscles to maintain balance.

The uncontrolled manifold (UCM) approach has been used to investigate joint coordination for COM control while standing [9], [10], [16], [17]. UCM analysis categorizes the joint combination variability into two components: V_UCM_ (combinations that allow for task flexibility) and V_ORT_ (combinations that cause unwanted task variable variability) [18]. V_UCM_ does not affect the COM position, whereas V_ORT_ leads to changes in the COM position. UCM analysis provides quantitative information on the relative proportion of joint variability [12], [16], [19], [20]. The UCM effect posits that the CNS organizes motor elements (joints) to achieve a target value for important task-related variable (COM position). Previous studies on manual pointing [20], [21], finger coordination [5], [22] and balance perturbation [11], [12] have shown that adaptive changes occur in the relative proportion of V_UCM_ to V_ORT_.

Motor abundance is an essential feature of adaptive control. When performing multiple tasks simultaneously, an increase in the use of joint motor abundance could preserve COM stability [23]. However, adaptive coordination patterns to immobilized joints must be examined to determine whether this flexible control scheme still exists. The main purpose of this study was to investigate the effect of artificially eliminating knee and lumbar-thoracic joint motions on postural control when the arms performed targeting tasks simultaneously in standing. This study hypothesized that altered postural coordination patterns are assembled adaptively and that this ability to use motor abundance decreases if some joint DOFs are unavailable.

## Methods

### Ethics Statement

This study adhered to the principles of the Declaration of Helsinki for human research. Both the Research Ethics Committee of National Taiwan University Hospital and the Research Institutional Review Board of the University of Delaware Human Subjects Review Board approved this study. Written informed consent was acquired from each participant.

### Participants

Twelve healthy young adults (6 males and 6 females) without any known neurological deficits, musculoskeletal conditions, or balance disorders participated in this study. The participants’ mean (±1 standard deviation (SD)) age, body mass, and height were 27.3 (±3.9) years, 66.9 (±8.8) kg, and 171.6 (±9) cm, respectively.

### Apparatus

**Figure 1:**
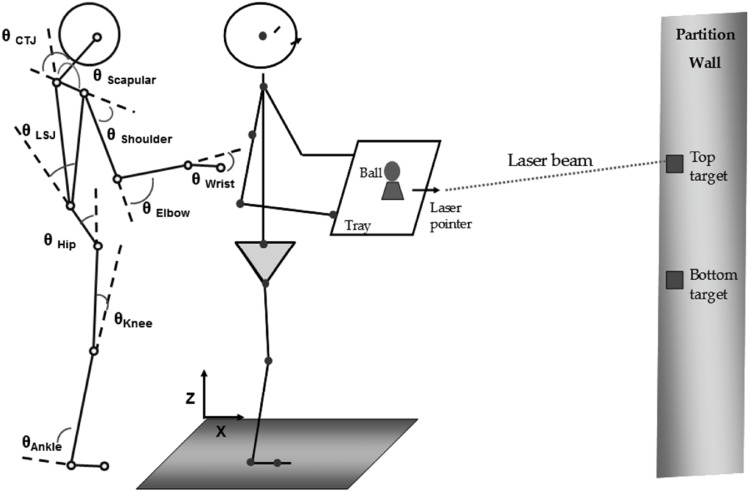
Experimental setup. Schematic illustration of the angles studied in the sagittal plane: ankle, knee, hip, lumbarsacral spine (LSJ), scapular, shoulder, elbow, wrist, and cervical-thoracic junction (CTJ). Note that the knee and LSJ were immobilized in the immobilized conditions.

A motion measurement system (Vicon, Oxford metrics, UK) recorded the motion of reflective markers placed on the right side of the body and trunk at 120-Hz. The experimenter carefully observed the participants’ movement and deemed them symmetrical. Spherical reflective markers, 1.5 cm in diameter, were applied to a tray and to various locations on the right side of the participant’s bodies with self-adhesive Velcro and hypoallergenic adhesive tape at the following locations (Figure 1): 1) the base of the 5th metatarsal; 2) the lateral malleolus; 3) the lateral femoral condyle; 4) the greater trochanter; 5) the lumbosacral junction (LSJ); 6) the cervical-thoracic junction (CTJ); 7) the zygomatic process of the temporal bone; 8) the acromion process of the shoulder; 9) the lateral humeral condyle; 10) the styloid process of the radius; and 11) the distal end of the third metacarpal bone.

Therefore, nine joint angles (DOFs) were available. The recorded coordinates from each reflective marker were filtered using MATLAB (Mathworks, Version 7.0.1, MA, USA) with a 5-Hz, low-pass bi-directional, 2^nd^-order Butterworth digital filter to eliminate artificial phase shifts in the data caused by filtering. Body segment lengths were derived from average marker positions during the first 3 s of the static standing trial.

### Experiment setup and procedures

The participants grasped each side of a lightweight tray with their hands (Figure 1). A small stand with a shallow indentation was firmly attached to the top surface of the tray. A laser pointer was firmly clamped to the front of the tray and pointed forward. It was used to point between 2 targets located on a wall partition in front of the participants. A racket ball was placed in the indentation on the small stand for combined targeting and ball-balancing tasks. The top and bottom targets on the wall partition, placed at a distance equal to each participant’s height, were set level with each participant’s acromion process and greater trochanter, respectively.

Two conditions (with and without joints constraints) with three levels of difficulty were established. The first level comprised a static standing task (STAT) with no arm movements. The participants were asked to stand quietly for 2 min, holding a tray without balancing a ball or pointing to targets. The second level–targeting with no ball (TNB)–required the participants to point back and forth between two 2.5-cm square targets mounted on a wall partition placed in front of them, with no additional ball-balancing tasks. A laser pointer mounted on the tray that participants held was used to point to the targets. The third level (targeting with ball-balancing, TWB) was similar to TNB; however, the participants were required to balance a racket ball on the tray they were holding and using for the targeting task.

All targeting tasks were paced with an auditory metronome. The metronome speed was the fastest speed at which the participant successfully performed the alternating targeting task during practice trials, while keeping a racket ball balanced on the stand mounted on the tray. No pause was allowed between pointing to the 2 targets. Participants were required to accumulate 80 successful reciprocal arm movement cycles without the ball falling. Once the ball dropped, the trial ended, and a new trial began. To minimize fatigue, a trial ended if a participant reached 40 consecutive successful cycles without the ball falling. Only trials with at least 10 consecutive cycles were included in the 80-trial count.

For the constrained conditions, the participants performed exactly the same tasks, except that they wore knee braces to restrict knee motion and a trunk orthosis to restrict thoracic and lumbar spine joint motion. Therefore, nine joint DOFs were available in the unconstrained conditions (STAT9, TNB9 and TWB9), and seven joint DOFs were available in the immobilized conditions (STAT7, TNB7 and TWB9).

### Data reduction

#### Time normalization of movement paths

The onset of upward targeting movement was selected based on the time of the occurrence of the minimal vertical position of the tray marker. The end of the upward excursion was selected based on the time of the maximal vertical position of the tray marker. Movement onset and cessation were confirmed using the vertical velocity profile of the same tray marker. The velocity at each onset and cessation event should be zero. Once the movement onset and cessation were determined, the data were cropped at those sample values and a cubic spline interpolation algorithm was used to normalize all movement paths to 100% (100 samples) in MATLAB.

#### Trial selection

To examine how motor abundance was used to control the COM position when the participants performed additional arm targeting tasks, trials were selected for analysis based on participant landing-point errors. Therefore, the mean of all laser pointer landing-point positions was calculated. Trials with landing-point positions within ±1 SD of the mean landing-point in the medial-lateral direction and within ±1 SD of the mean landing-point in the vertical direction were included. A comparison algorithm written in MATLAB was used to identify trials that met both criteria. This resulted in an average of 29.5 cycles per condition for analysis (TWB9: 31.5 ± 6.2 cycles; TNB9: 30.2 ± 6.1 cycles; TWB7: 28.5 ± 4.4 cycles; and TNB7: 28.1 ± 4.0 cycles.).

### Dependent variables

#### Joint angle

**Figure 2:**
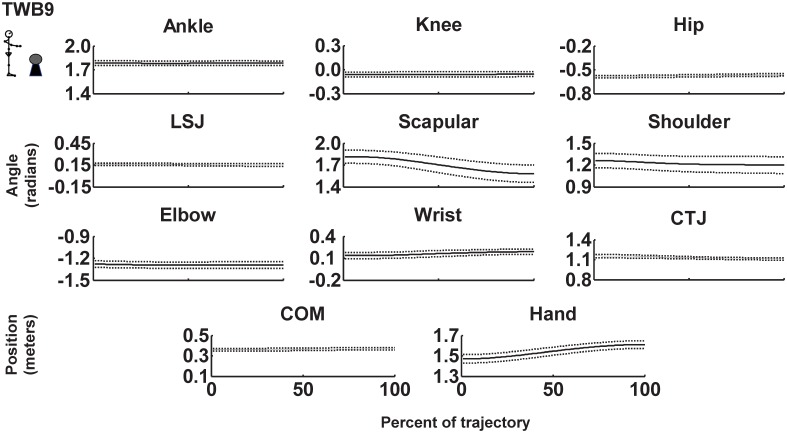
Mean trajectory of joints, center of mass (COM), and hand mean trajectories with respect to the coordinate system origin for a representative participant when targeting a small target and balancing a ball in the unconstrained condition (TWB9). Dashed lines represent ±1 SD from the mean. LSJ: lumbo–sacral junction. CTJ: cervical-thoracic junction.

**Figure 3:**
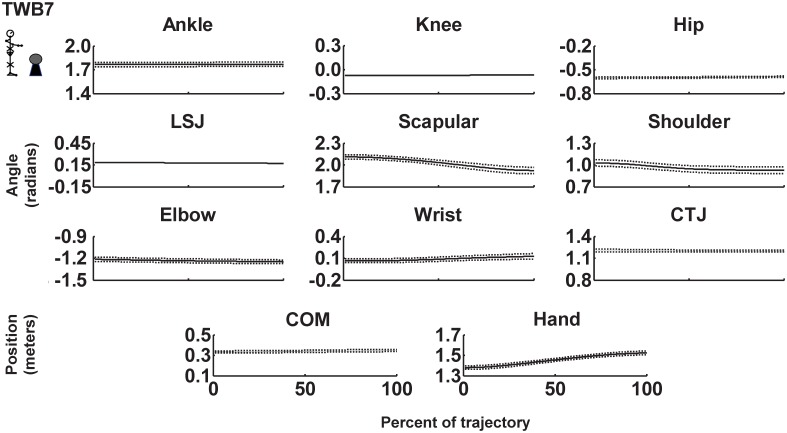
Mean trajectory of joints, center of mass (COM), and hand with respect to origin of the coordinate system for a representative participant in the targeting to a small target and combining with the ball-balancing in the immobilized condition (TWB7). Dashed lines represent ±1 SD from the mean position. LSJ: lumbo–sacral junction. CTJ: cervical-thoracic junction.

Reflective marker coordinates were used to calculate sagittal plane joint angles: (1) ankle, (2) knee, (3) hip, (4) lumbo–sacral junction (LSJ), (5) sternal-clavicular (6) shoulder, (7) elbow, (8) wrist, and (9) cervical-thoracic junction (CTJ). In the immobilized condition, immobilized joint angles were substituted with constants obtained from average immobilized joint angles across cycles for a given condition. The general formula used to calculate joint angles was:
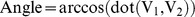
where *V_1_* is the unit vector representing the proximal segment, and *V_2_* is the unit vector representing the distal segment. Joint variability was calculated using the joint variance at each percentage of normalized movement time. Because variability was relatively stable across different cycle percentages (see Figure 2 and Figure 3), it was then averaged over all upward movements.

#### Center of mass

The location of whole body COM was estimated at each point in time using measured body segment lengths, estimated locations of each segment’s COM along those lengths, and their proportion to the whole body COM [24]. The COM position was calculated from these segments and joint angles at a point in time using.
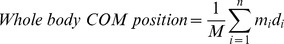
where 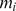 is the mass for each segment, 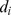 is the distance from one end of the segment to the segment COM, and 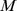 is the body weight of the participant. The whole body was divided into the feet, shanks, thighs, pelvis, trunk, scapulars, upper arms, forearms, hands, and head segments for the whole body COM calculation. Therefore, *n* = 10 in the COM model. Masses were doubled for lower limb segments. Whole body COM variability was calculated using the whole body COM variance at each percentage of normalized movement time. Because variability was relatively stable across different cycle percentages, it was then averaged over all upward movements.

#### UCM analysis

The UCM approach was used to separate joint configuration variability into two components to examine how supra-postural tasks affect the use of motor abundance to stabilize the COM position [18], [23]. The UCM effect is that the control system selects a subspace or manifold from the motor elements space (here joint posture space) that corresponds to a particular value for a task-related variable it is attempting to stabilize the COM position. In the context of this study, the UCM subspace is composed of all joint postures combinations with the same average COM position across cycles at each upward movement percentage. Therefore, joint posture variations within this subspace lead to a consistent COM position across cycles. By contrast, joint variations in the subspace orthogonal to the UCM, its complement, cause cycle-to-cycle variations in the COM position from its mean value. The extent of COM position stabilization, can then be examined by comparing two types of variances, within the UCM subspaces (V_UCM_) and orthogonal subspaces (V_ORT_). When V_UCM_ is substantially greater than V_ORT_, the CNS organizes motor elements (joints) to achieve a target value for an important task-related variable (COM position).

During voluntary movements, the formal relationship between motor element variables changes and task level changes (i.e., the Jacobian matrix) varies according to limb configurations. In this case, UCM analysis is performed at the same trajectory point in multiple cycle repetitions. Previous studies have extensively described the mathematical model used for UCM analysis [23], [25], and detailed computational procedures are listed below:

1. The first step inUCM analysis is to obtain a geometric model relating the task-related variable (COM position) to the elemental variables (joint angle configuration). Joint configuration is composed of 9 angles: (1) ankle, (2) knee, (3) hip, (4) the LSJ, (5) scapular, (6) shoulder, (7) elbow, (8) wrist, and (9) CTJ. Therefore, 9 joint configuration space dimensions are used for hypotheses on anterior-posterior COM position control. We focused on an analysis of joint variability with respect to COM position control, which spans a 1 dimensional task space. The geometric model relating COM position to joint configuration, with the origin at the foot, is.

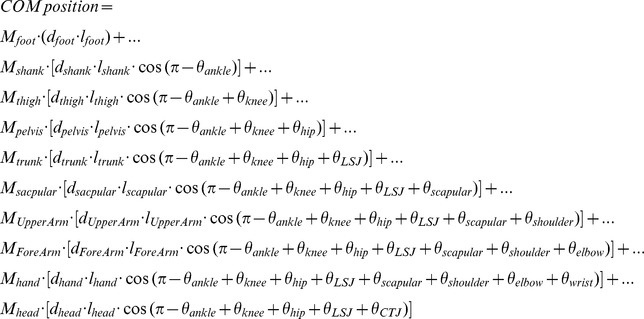
where {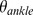
…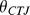} are the externally defined joint angles; {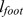
…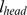} are the respective segments lengths calculated in the static calibration trial; {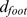
…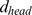} are the segment length percentages from the distal end where the mass of the segment lies; and {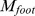
…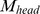} are the proportions of the total body mass of each segment [24].

1. Small changes in the task-related variable are related to changes in joint angles *(θ)* through the Jacobian matrix, *J*, which is a matrix of partial derivatives matrix of the task-related variable (i.e., *COM*) relative to joint angles. The Jacobian matrix is:
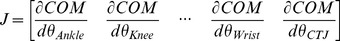

2. The null-space of the Jacobian matrix was obtained using the mean joint configuration of all movement cycles at each normalized time point. This linear subspace represents all joint deviations from the mean joint configuration that resulted in no changes in the COM position.
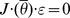

where 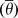 is the mean joint configuration and 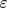 are null-space vectors.

1. The mean-free joint configuration at each time point was then projected onto the null-space and its complement (i.e., the orthogonal subspace):
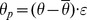


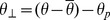

where 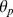 is a vector of joint configuration projected onto the null-space and 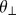 is a vector of joint configuration projected orthogonally onto the null-space (linear approximation of the UCM).

1. The variance of the projections into each dimension of the 2 subspaces was then computed and divided by the number of dimensions in each space:
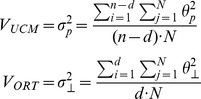

where 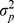 is variance within the UCM (V_UCM_); 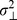 is variance in the joint space orthogonal to the UCM (V_ORT_). N is the number of repetition (cycles), n is the total number of DOFs (joints), and d is the number of DOFs describing the task. Because the UCM and orthogonal space dimensions are different, the variance components computed for each subspace were normalized to the number of dimensions of the respective subspace. Thus, the V_UCM_ and V_ORT_ are the variances per DOF. In dynamic targeting tasks, for example, the task space is one-dimensional and the UCM is an eight-dimensional subspace (9–1 = 8) for the COM position stabilizing hypothesis. Therefore, the projection variances within the UCM were divided by 8, and the projection variances orthogonal to the UCM were divided by 1.

For dynamic targeting tasks, V_UCM_ and V_ORT_ calculations were performed at each percentage across time-normalized movement cycles. The results were then averaged across the cycle for statistical analysis. For the static standing condition, V_UCM_ and V_ORT_ were calculated over the entire 2 min of quiet stance.

However, in the immobilized condition, the V_UCM_ and V_ORT_ dimensions were different from those in the unconstrained condition. This study hypothesized that the COM position was stabilized using combinations of the 7 joint motions, the task space was 1-dimensional subspace, and the UCM was a 6-dimensional subspace (7–1 = 6). Therefore, the variance of projections within the UCM (V_UCM_) was divided by 6, and the variance of projections orthogonal to the UCM (V_ORT_) was divided by 1.

### Statistical analyses

A paired *t*-test was performed to compare the combined task failure rate between the unconstrained and immobilized conditions (TWB9 vs. TWB7). A repeated-measures 2-way ANOVA (immobilized conditions (9-DOFs vs. 7-DOFs) x tasks (STAT vs. TNB)) was performed to evaluate the main effect differences between independent variables. Another repeated measure two-way ANOVA (immobilized conditions (9-DOFs vs. 7-DOFs) x tasks: (TNB vs. TWB)) was performed to evaluate the main effect differences between independent variables. Two 3-way repeated-measures ANOVA (First: variance components (V_UCM_ vs. V_ORT_) x tasks (STAT vs. TNB) x immobilized conditions (9-DOFs vs. 7-DOFs); Second: variance components (V_UCM_ vs. V_ORT_) x immobilized conditions (9-DOFs vs. 7-DOFs) x targeting conditions (TNB vs. TWB)) were used to determine how tasks differences affect motor abundance use, which was indexed by V_UCM_ and V_ORT_ changes with respect to COM control.

Significant interactions were further analyzed using Bonferroni pairwise comparisons. All statistical analyses were performed with SPSS v.16.0 software (SPSS, Inc., Chicago, IL, USA) and the α-level was set at 0.05.

## Results

### Ball-balancing task success error

The analysis performed on the ball-balancing task success error attempted to prove that task difficulty was the same for the participants, ruling out the confounding variable of task complexity. The failure rate (dropping the ball) of the combined task (TWB9) was 2.35±1.71% (mean ± SD) in the unconstrained condition and 2.38±1.18% in the immobilized condition (TWB7). No significant difference between the 2 immobilized conditions was found (TWB9 vs. TWB7: *t* = −0.38, *p* = 0.97).

### Joint (motor elements) variability

**Figure 4:**
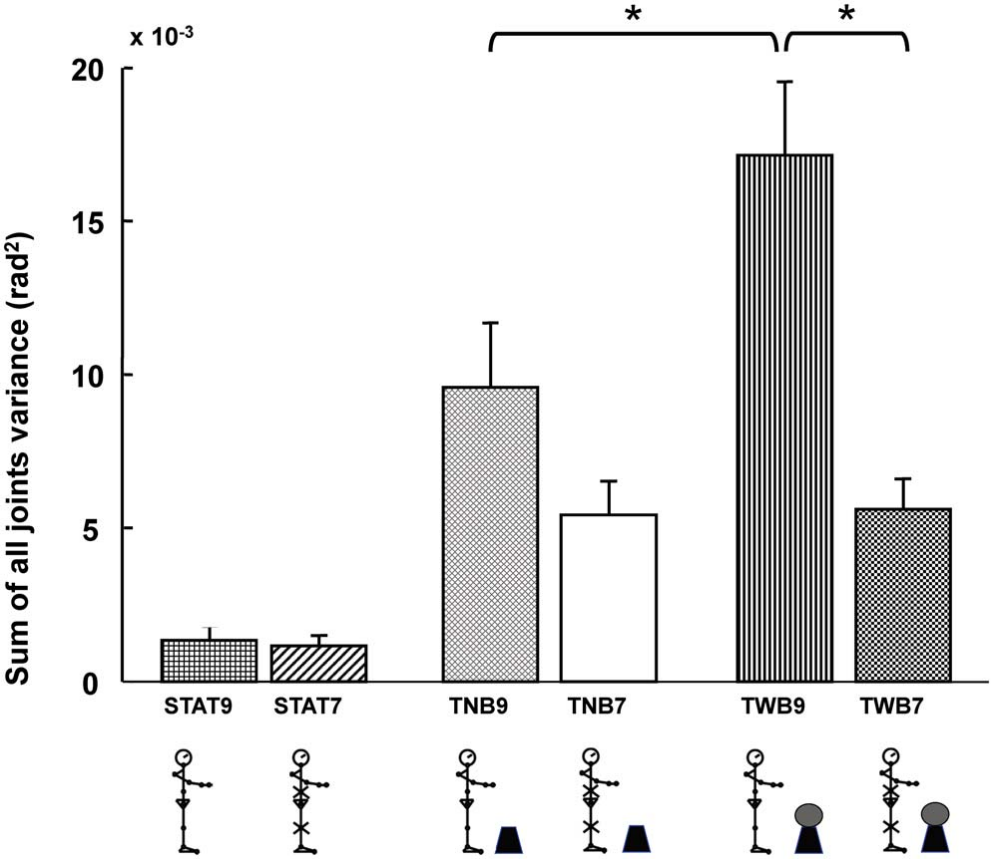
Mean (+SEM) of the sum of all joints variability across values of all participants. The bars represent: the static standing task in an unconstrained condition (STAT9), static standing task in an immobilized condition (STAT7), targeting task with no ball balancing in an unconstrained condition (TNB9), targeting task with no ball-balancing task in an immobilized condition (TNB7), targeting task combined with the ball-balancing task in an unconstrained condition (TWB9), and targeting task combined with the ball-balancing task in an immobilized condition (TWB7).

Figure 2. and Figure 3 show the mean ± SD joint positions across in all cycles for targeting and combined targeting and ball-balancing tasks for a representative participant for the unconstrained (TWB9) and immobilized conditions (TWB7), respectively. Compared to unconstrained joints, joint variability decreased when joints were immobilized for this representative participant. **Table 1** lists the average variability of each joint, and Figure 4 shows the sum of all joint variabilities. The sum of all joint variabilities was significantly less in the static standing task than in the targeting task, regardless of immobilized condition (*F*_1,11_ = 22.59, *p*<0.001). However, the immobilized condition showed no significant main effect (*F*_1,11_ = 2.13, *p* = 0.17), independent of the arm task, and there was no interaction between the immobilized condition and arm task (*F*_1,11_ = 2.20, *p* = 0.16).

**Table 1:** Variability of individual joint during two minutes standing.

Joint	STAT9	STAT7	TNB9	TNB7	TWB9	TWB7
Ankle	0.03±0.01	0.03±0.01	0.16±0.07	0.08±0.03	0.19±0.07	0.14±0.06
Knee	0.05±0.02	0	0.15±0.07	0	0.44±0.14	0
Hip	0.03±0.01	0.03±0.01	0.07±0.01	0.05±0.02	0.36±0.27	0.17±0.03
LSJ	0.08±0.02	0	0.15±0.03	0	0.19±0.06	0
Scapular	0.19±0.05	0.22±0.07	1.18±0.42	0.80±0.20	3.46±1.02	0.79±0.18
Shoulder	0.20±0.05	0.31±0.10	1.89±0.59	1.30±0.33	4.12±0.99	1.36±0.26
Elbow	0.27±0.13	0.15±0.08	2.50±1.17	1.66±0.49	5.24±1.38	1.66±0.56
Wrist	0.36±0.17	0.18±0.08	3.25±1.41	1.30±0.49	3.28±0.63	1.08±0.18
CSJ	0.13±0.03	0.22±0.07	0.50±0.11	0.40±0.11	0.34±0.07	0.67±0.43

Condition acronyms are the same as in Figure 4 (Units: 10^−3^ radian^2^; the mean± SEM for all participants are shown). LSJ: lumbo–sacral junction. CTJ: cervical-thoracic junction.

When comparing the combined arm tasks to the targeting task alone, significant main effects were found for the immobilized condition (*F*_1,11_ = 12.72 *p*  = 0.004) and arm task (*F*_1,11_ = 14.23, *p* = 0.003). A significant interaction (*F*_1,11_ = 12.45, *p* = 0.005) resulted from an overall joint variability decrease in TWB7, compared to TWB9 (*F*_1,11_ = 18.80, *p* = 0.001). The joint variability decrease in TNB7, compared to TNB9, was not significant (*F*_1,11_ = 2.20, *p* = 0.17).

### COM position (task-related variable) variability

**Figure 5:**
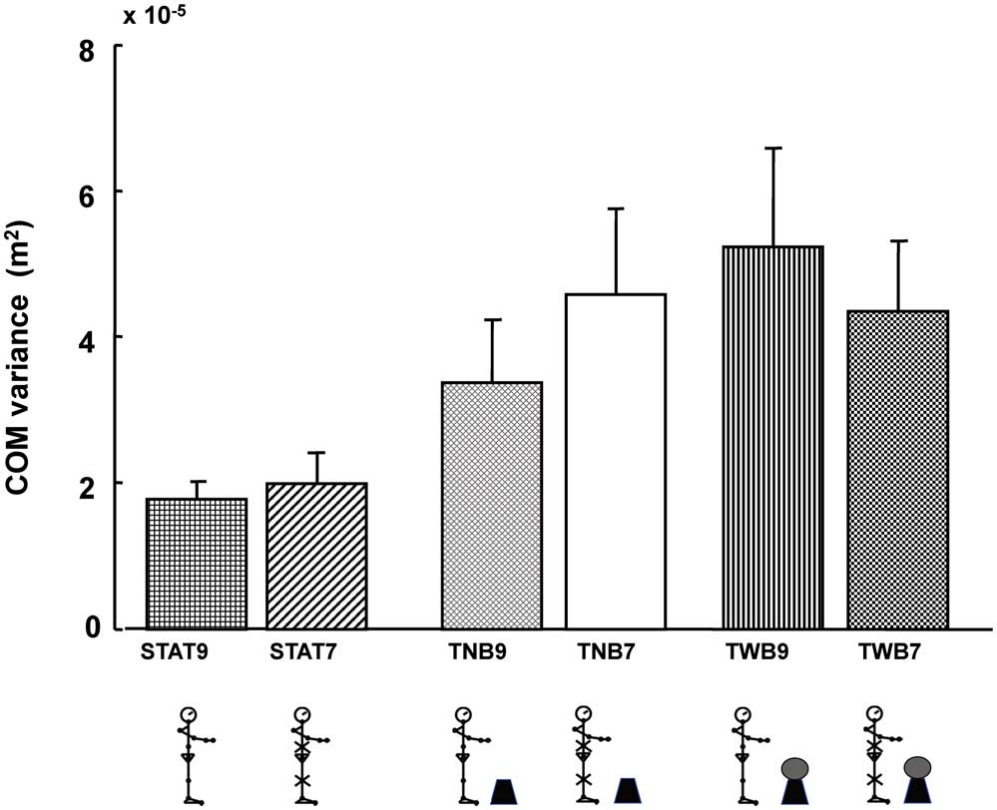
Mean (+SEM) of task-related variable variability in the anterior-posterior movement direction for center of mass (COM) position. Condition acronyms are the same as in Figure 4.

Figure 5 shows the means and standard error measurements (SEMs) of the anterior-posterior movement direction for the COM and hand position calculated over all cycles.

When comparing STAT9, STAT7, TNB9, and TNB7 conditions, COM position variability was not significantly different for the immobilized conditions, independent of the arm task (F1,11 = 1.52, p = 0.24). However, COM position variability was significantly higher in the targeting task than in the static standing task, independent of immobilized condition (*F*_1,11_ = 7.67, *p* = 0.02).

When comparing COM position variability among conditions TNB9, TNB7, TWB9, and TWB7, there were no significant main effects for the immobilized condition (*F*_1,11_ = 0.03, *p* = 0.85), arm task (*F*_1,11_ = 1.24, *p* = 0.28), nor did any interaction exist between these factors (*F*_1,11_ = 2.04, *p* = 0.18). These findings indicate that immobilizing joint motions does not significantly affect COM position variability.

### Structure of joint configuration variability for COM position control (UCM analysis)

**Figure 6:**
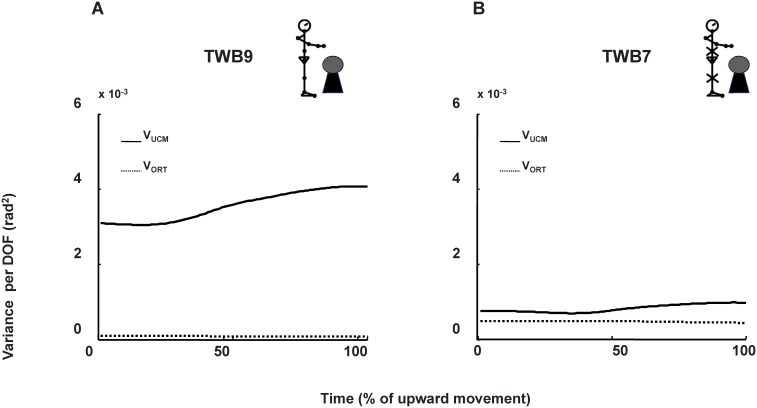
V_UCM_ and V_ORT_ with respect to the COM control for a representative participant. A: Targeting combined with ball-balancing task in an unconstrained condition (TWB9). B: The same task with immobilized joints (TWB7). Solid lines represent the mean V_UCM_of all cycles and dashed lines represent the mean V_ORT_ of all cycles.

**Figure 7:**
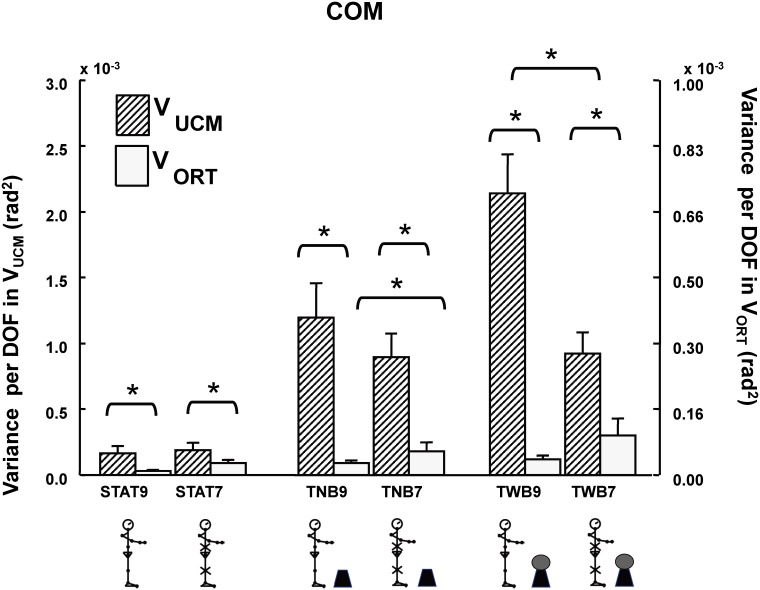
Mean (+SEM) of joint configuration variability for COM position control. Adjacent pairs of bars represent V_UCM_ (left, diagonal filled bars) and V_ORT_ (right, spotted bars). Condition acronyms are the same as in Figure 4. The scale of V_UCM_ is larger than the scale of V_ORT_, however, the relationship between V_UCM_ and V_ORT_ did not change, that is, V_UCM_>V_ORT_. Asterisks indicate significant differences between bars (*p<0.05).

Figure 6 depicts the raw UCM result for a representative participant. Figure 7 shows the assembled averages for the results of decomposing joint configuration variability into V_UCM_ and V_ORT_ with respect to the COM control for all participants. When V_UCM_>>V_ORT_, this is referred to as the “UCM effect” (see Methods).

Quiet stance was the lowest level of difficulty. When participants stood quietly, V_UCM_, which represents the joint combination variability equivalent to the stabilized COM position, was substantially larger than V_ORT_, which represented the joint combination variability that tended to change COM position (STAT9: *F*_1,11_ = 8.66, *p* = 0.01; STAT*7*: *F*_1,11_ = 9.79, *p* = 0.01). This UCM effect, i.e., V_UCM_ >>V_ORT,_ was apparent for all testing conditions. For the comparison between conditions, when joints were immobilized (STAT7), V_UCM_ did not differ from the unconstrained condition (STAT9) (V_UCM_: *F*_1,11_ = 0.53, *p* = 0.48). Although V_ORT_ tended to be large when joints were artificially eliminated, the difference was insignificant (V_ORT_: *F*_1,11_ = 3.70, *p* = 0.08).

Targeting task was the second level of difficulty. When participants independently performed the targeting task (TNB9 and TNB7), the UCM effect was also apparent (TNB9: *F*_1,11_ = 20.27, *p* = 0.001; TNB7: *F*_1,11_ = 24.30, *p*<0.001), indicating that most joint combination variability was structured selectively to stabilize the COM position. However, when the immobilized conditions for V_UCM_ and V_ORT_ were separately considered, V_ORT_ significantly increased in TNB7 (compared with TNB9; *F*_1,11_ = 4.64, *p* = 0.05.), whereas V_UCM_ was the same for the immobilized and unconstrained conditions (*F*_1,11_ = 1.87, *p* = 0.20).

Combining ball-balancing and targeting tasks was the highest level of difficulty. When the ball-balancing and targeting tasks (TWB9 and TWB7) were combined, the UCM effect remained apparent (that is, V_UCM_>V_ORT_; TWB9: *F*_1,11_ = 39.27, *p*<0.001; TWB7: *F*_1,11_ = 28.26, *p*<0.001). However, V_UCM_ decreased significantly (*F*_1,11_ = 24.9, *p*<0.001) and V_ORT_ tended to increase (F1,11 = 3.13, p = 0.06) when joints were immobilized, compared with when joints performed freely. Table 2 summarizes the V_UCM_ and V_ORT_ differences between immobilized conditions for various targeting tasks”.

**Table 2:** Difference between conditions in V_UCM_ and V_ORT_ (unit: Units: 10^−3^ radian^2^ per DOF).

Comparison	Variance	COM
STAT9 minus STAT7	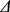 V_UCM_	−0.02
	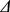 V_ORT_	−0.01
TNB7 minus STAT7	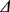 V_UCM_	0.71
	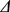 V_ORT_	0.03
TNB9 minus TNB7	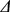 V_UCM_	0.30
	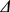 V_ORT_	−0.04
TWB9 minus TWB7	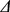 V_UCM_	1.22
	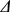 V_ORT_	−0.06

Condition acronyms are the same as in Figure 4.

## Discussion

This study investigates the effect of artificially eliminating knee and lumbar-thoracic joint motions on postural control when the arms are used to perform targeting tasks simultaneously in standing. The results supported our hypothesis that reflect an altered adaptive coordination pattern when the knee and trunk are immobilized. That is, the ability to use motor abundance decreases when joint DOFs are unavailable. This study provides further confirmation to determine the role of motor abundance in facilitating multitask performance.

### Immobilized joints resulted in a less flexible joint coordination pattern during multi-tasking performance

In both arm tasks, the use of motor abundance decreased when the joints were immobilized. Constraint of the knee, lumbar and thoracic spine joints while performing the targeting task alone resulted in significant increased V_ORT_ and slightly decreased V_UCM,_ compared to the unconstrained condition. When combining the targeting and ball-balancing task (multiple tasks), V_UCM_ decreased significantly and V_ORT_ increased slightly when joints were immobilized. However, both arm tasks did not appear to adversely affect the ability to stabilize the COM as predicted. It is likely that although the number of available DOFs were reduced, the nervous system had a sufficient numbers of DOFs available to stabilize the COM for interactive moments created by arm movement tasks in a motor-equivalent manner using a less flexible pattern (i.e., decreased V_UCM_ and increased V_ORT_). Motor equivalence is the ability to use different effectors (i.e., muscles and joints) to achieve the same desired motor goal, despite intentional or imposed changes in the number of body segments involved in a task [9], [25]–[27].

When participants stood quietly without performing an additional task, joint variability representing joint combinations that were equivalent with respect to stabilize the COM (i.e. V_UCM_) was significantly higher than V_ORT_, regardless of the immobilized condition. This result is consistent with the results of previous studies [11], [23], and has been referred to as the UCM effect. Although V_ORT_ and COM position variability increased slightly in the immobilized condition compared to the unconstrained condition when standing quietly, these increases were not significant. Therefore, when the participants stood quietly without performing an additional task, reducing the available joint DOFs had a minimal impact on COM control. By contrast, when performing multiple tasks simultaneously with reduced available joint DOFs, the participants were forced to use a less flexible control for COM.

Using a less flexible control scheme could be problematic. A previous study investigated the effect of an ankle brace on knee and hip joints during trunk rotation tasks when standing [28]. The results showed reduced trunk axial rotation during ball catching tasks and increased knee axial rotation during target touching tasks. This adaptive control strategy increased knee joint stress and may increase knee injury risk. An experimental study also observed increased postural sway from joint immobilization at the ankle, knee, hip, and trunk [29]. Without all available joint DOFs, the body may not be able to take advantage of motor abundance to control the upright balance and result in a detrimental movement strategy.

### Sensory reweighting and cognitive perception affected adaptive control

The braces and orthoses used in this study may also have affected the results. Anecdotally, some participants reported that the braces provided legs and body support, facilitating the targeting task. Consequently, they could focus more on performing the arm targeting movement. It is possible that the braces and orthoses in this study provided contact cues for the somatosensory system. Studies have demonstrated that somatosensory contact cues affect postural sway. Jeka and colleagues found that even light touch contact cues from the fingertip are providing information about body that can be used to reduce postural sway through postural muscle activation [30], [31]. Future studies could evaluate subjective reports using questionnaires or visual analogue scales to confirm the supportive effect of the braces.

Cognitive mechanisms may also affect the postural control scheme. If participants felt more stable when wearing the braces and orthoses, this may have perceptually affected their control strategy. Slijper and Latash concluded that postural adjustments show changes associated with the mechanical and perceptual aspects of a task [32]. In their study on fast arm movement performed by standing participants, anticipatory postural adjustments in the leg and trunk decreased significant with an added finger touch. These adjustments did not change when the touch was substituted with a hand grasp. Therefore, the nervous system may treat a finger touch (or brace contact in this study) as a stabilizing factor and change the properties of sensory reweighting to control upright stance [33].

Adaptive goal-directed behavior in humans relies on the integration of dorsal and ventral streams. The execution of a goal-directed action depends on the control systems in the dorsal stream, whereas the selection of suitable goal objects and the action to be performed depends on the perceptual part of the ventral stream [34]. The dorsal stream mediates the visual control of skilled actions by registering visual information about the goal object on a moment-to-moment basis, converting this information into applicable coordinates for the effector being used [35]. The dorsal stream does not utilize the high-level perceptual representations of the object assembled by the ventral stream, and instead depends on current bottom-up information from the retina to identify the required movement parameters [35]. What, then, accounts for the decreased use of motor abundance to stabilize the COM when joints were constrained? One possible explanation is a decrease in the feedback loop gains. Previous studies have shown that the nervous system uses tunable back-coupling via feedback loops to compensate for errors among movement components in a task-specific way [36], [37]. The control system adjusts gains from central feedback loops among neurons, the output of which specifies the values of elemental variables. Motor abundance possibly enhanced these feedback loops and was compromised when joints were constrained.

### Implications and future directions

The recruitment-suppression hypothesis of the freezing and freeing of DOFs supports the notion that increased joint activity can lead to greater environmental adaptation [38]. An ample range of joint combination variability gives the body the freedom to adopt orientations, configurations, and movements that allow the generation of exploratory variation in postural behavior [23], [39]. Increased joint activity may also enhance participant perceptions of ongoing postural configurations as an increase in feedback loops gains. This study provides evidence that during multi-task performance, intersegmental coordination was more flexible when all joints were available, as in the usual situation of a healthy human. In contrast, immobilized joints resulted in a decreased use of motor abundance and compromised the error-compensation ability among the joints.

Although this flexible pattern did not directly benefit the control of task-related variable when joints were immobilized, this may be due to the task not having sufficed in challenging to reveal task level differences or because the flexible pattern was used to stabilize other task-related variables. It is uncertain why the nervous system would select a control strategy may consume more energy if the joint combination range used was simply a reflection of system noise. Future studies should investigate different sources that could be of benefit from motor abundance. Brain cortical activity and postural muscle activity responses associated with multi-tasking could be measured. For example, the attention demands of the task may decrease when the nervous system exploits motor abundance. Studies have shown that specific neural activation patterns in electroencephalography (EEG) [40] and functional magnetic resonance imaging [41] were associated with recognition of unstable postures in young healthy participants.

Postural reactions to multi-tasking perturbation may be triggered by central command mechanisms identified by a burst of EEG gamma activity or induced activation of distinct areas of the brain including the bilateral parietal cortex, anterior cingulate cortex, and bilateral cerebellum. Tests can determine whether brain activity changes when the nervous system exploits motor abundance during multi-tasking. Similar observations could be conducted at the muscle level. Studies have developed highly complex computational methods of reducing muscle activation patterns to identify a more simplified synergy organization, bringing new insights to the DOF problem in postural control [42], [43]. The existence of motor abundance use at the muscle level during multi-tasking should also be validated.

### Conclusions

This study investigated postural control adaptation joint immobilization during multi-task performance. The findings confirm that motor abundance provides an advantage to the CNS when performing multiple tasks simultaneously. Despite the limited significance of the results in the predicted direction; that is, V_UCM_ decreased and V_ORT_ increased when the joints were artificially immobilized. Future studies should examine how people with natural joint limitations such as patient populations can stabilize their COM when performing similar arm movement tasks. An understanding of fundamental deficits or adaptations during the multiple task performance while standing is important for development of rehabilitation approaches.
